# Long Noncoding RNA LL35/Falcor Regulates Expression of Transcription Factor Foxa2 in Hepatocytes in Normal and Fibrotic Mouse Liver

**DOI:** 10.32607/20758251-2019-11-3-66-74

**Published:** 2019

**Authors:** O. V. Sergeeva, S. A. Korinfskaya, I. I. Kurochkin, T. S. Zatsepin

**Affiliations:** Skolkovo Institute of Science and Technology, Bolshoy Blvd. 30, bldg. 1, Moscow, 121205, Russia; Department of Chemistry, Lomonosov Moscow State University, Leninsikie gory 1, bldg. 3, Moscow, 119991, Russia

**Keywords:** non-coding RNA, transcription factor Foxa2, regulation, liver

## Abstract

Long noncoding RNAs (lncRNA) play important roles in the regulation of transcription, splicing, translation, and other processes in the cell. Human and mouse lncRNA (DEANR1 and LL35/Falcor, respectively) located in the genomic environment in close proximity to the Foxa2 transcription factor were discovered earlier. In this work, tissue-specific expression of LL35/Falcor lncRNA has been shown in mouse liver and lungs. The use of antisense oligonucleotides allowed us to achieve LL35/Falcor lncRNA downregulation by 90%. As a result, the level of Foxa2 mRNA and protein dropped, which confirms the involvement of LL35/Falcor lncRNA in the regulation of transcription factor Foxa2. We have shown a decrease in the expression of LL35 lncRNA in liver fibrosis, which correlates with the previously published data for mRNA Foxa2. Thus, lncRNA LL35 regulates Foxa2 expression in the liver not only in normal conditions, but also during development of fibrosis, which allows one to consider lncRNA a biomarker of this pathological process.

## INTRODUCTION

Analysis of the human transcriptome demonstrated that less than 2% of the genome encodes proteins, while noncoding RNA genes prevail among the remaining 98%. Among the variety of noncoding RNAs, short (less than 200 nucleotides in length) and long RNAs (more than 200 nucleotides) can be distinguished [1]. Long noncoding RNAs (lncRNAs) perform regulatory functions in all major cellular processes. They participate in the regulation of transcription both locally (in cis) and remotely (in trans), having an impact on such regulatory elements as promoters and enhancers, as well as the chromatin structure and RNA polymerase activity [2]. LncRNAs can participate in the regulation of translation [3] and alternative splicing by recruiting protein factors [4], serve as “molecular sponges” for miRNAs, and regulate their level of free form in the cell [5]. LncRNAs are also often expressed in tissue-specific manner and/or transcribed only in certain conditions. An uncontrolled increase of lncRNAs transcription, such as MALAT-1, HOTAIR, H19, and HULC, stimulates the development of oncological diseases [6].

The number of characterized functionally important long noncoding RNAs increases every year. However, their mechanisms of action remain unknown for the most part. Despite the fact that cell culture studies allow us to describe the molecular mechanisms of lncRNA action, the use of animal models provides a more general and consistent approach to the study of lncRNA functions. However, the low homology between lncRNAs even amongst closely related species complicates such studies and, in some cases, the homology is observed only at the level of the secondary structure. The search for functional analogues of human lncRNAs in mice also allows us to expand the conditions for a functional study of lncRNAs by using various mouse disease models.

It has been previously shown that human lncRNA DEANR1 regulates the proliferation and promotes the apoptosis of choriocarcinoma cells [7], it influences the Notch signaling pathway [8], and serves as a potential biomarker for a number of cancers such as choriocarcinoma [9], gastric [10], pancreas [11] and colon [12] cancers, as well as several types of lung cancer [13]. The DEANR1 lncRNA gene is located in close proximity to the Foxa2 transcription factor gene in the human genome. DEANR1 is involved in the regulation of Foxa2 during the differentiation of human pancreatic endoderm cells [14]. The authors proposed a mechanism for the activation of Foxa2 transcription through the recruitment of Smad2/3 proteins to its promoter by DEANR1. Foxa2 is essential for liver development from the endoderm [15] and serves as a transcriptional activator of the liver-specific genes that encode albumin and transferrin. Foxa2 also plays an important role in glucose homeostasis in the liver [16]. An analysis of the genomic environment of Foxa2 in the mouse genome revealed a potential functional analogue of DEANR1: LL35/Falcor lncRNA (hereinafter referred to as LL35) [17]. Knockout of the gene encoding LL35 lncRNA leads to a decrease in the level of Foxa2 mRNA by 25-30% in the mouse lung epithelium and does not result in a pronounced phenotype in the development of embryo lungs. However, LL35 plays an important role in the regulation of Foxa2 in response to additional exposure, such as lung damage [18]. In this work, LL35 lncRNA has been characterized: we established its tissue-specific expression in mouse organs and demonstrated its intracellular localization. We compared different approaches to lncRNA knockdown, achieved LL35 lncRNA knockdown, and demonstrated the involvement of Foxa2 transcription factor in mouse hepatocytes. Also, we revealed a drop in the level of LL35 lncRNA in liver fibrosis.

## EXPERIMENTAL

**Cell lines**

AML12 mouse hepatocytes (ATCC, USA) were cultured in a DMEM/F12 medium supplemented with 10% fetal bovine serum at 37°C and 5% CO_2_.

**Isolation of nuclear and cytoplasmic cell fractions**

AML12 cells (~1 × 10^6^) were grown under standard conditions, then they were removed from the substrate using a 0.25% trypsin solution in 0.5 mM EDTA, washed with phosphate buffer (10 mM sodium phosphate, 100 mM sodium chloride, pH 7.4), followed by centrifugation at 500 g for 5 min. The pellet was resuspended in CE buffer (20 mM HEPES (pH 7.4), 1.5 mM MgCl_2_, 10% glycerol, 0.05% NP-40) containing protease inhibitors; incubated on ice for 10 min; and centrifuged at 1,700 g and 4°C for 5 min. The supernatant fraction contained the cytoplasmic cell extract. The pellet was resuspended in NE buffer (20 mM HEPES (pH 7.4), 1.5 mM MgCl_2_, 10% glycerol, 0.05% NP-40, 500 mM NaCl) containing protease inhibitors; incubated on ice for 10 min; and centrifuged at 1,700 g for 5 min at 4°C. The supernatant fraction contained the nuclear cell extract. Total RNA was isolated from the separated extracts using the Trizol reagent (Invitrogen, USA) according to the manufacturer’s protocol.

**RT-qPCR**

Total RNA was isolated from mouse organs or AML12 cells using the Trizol reagent (Invitrogen, USA) according to the manufacturer’s protocol. Next, ~1 μg of total RNA was treated with DNase I (Thermo Scientific, USA) according to the manufacturer’s instructions in order to remove residual genomic DNA. Reverse transcription was performed using a Maxima First Strand cDNA synthesis kit (Thermo Scientific, USA). The reaction mixture was 3x diluted with water, and qPCR was performed using a PowerUp SYBR Green Master Mix reagent kit (Applied Biosystems, USA) according to the manufacturer’s protocol (0.3 μM primer mixture, 0.2 μg of cDNA). For the amplification of LL35 lncRNA, two sets of primers were selected which were further used in all experiments (table). The reaction products were analyzed by 1% agarose gel electrophoresis in TAE buffer (40 mM Tris-acetate, 1 mM EDTA, pH 7.6). For RT-PCR of the isolated cell fractions, the level of target RNA in the nuclear fraction was normalized to the level of U2 snRNA and the level of target RNA in the cytoplasmic fraction was normalized to Gapdh mRNA.

**Table 1:** Primers for RT-qPCR, siRNA, and ASO used in the study

Primer	Nucleotide sequence, 5’→3’
LL35-1-F	TTTGGCCAAGGGAGAAAGCTCAGA
LL35-1-R	ACGGTGCCTGTAACTTACCTGAAG
LL35-2-F	GCTCGGTTTGAGCTCAAATAAATG
LL35-2-R	CAGAGGCTCTAGCCACGATGGAG
Gapdh-F	TGCACCACCAACTGCTTAGC
Gapdh-R	GGATGCAGGGATGATG
U2-F	GAAGTAGGAGTTGGAATAGGA
U2-R	ACCGTTCCTGGAGGTA
Foxa2-F	TATGCTGGGAGCCGTGAAGATGG
Foxa2-R	GCGTTCATGTTGCTCACGGAAGAG
siRNA 1	cucAAAGuuuAGAGuucAuTsT AUGAACUCuAAACUUUGAGTsT
siRNA 2	uAAcuuAccuGAAGAGGAATsT UUCCUCUUcAGGuAAGUuATsT
siRNA 3	cuGAAuuAGAGAAAcAAcuTsT AGUUGUUUCUCuAAUUcAGTsT
siRNA 4	GucAGuAAAcAAccGAAAATsT UUUUCGGUUGUUuACUGACTsT
siRNA 5	GuGGAAuAAuGuuAAGcuuTsT AAGCUuAAcAUuAUUCcACTsT
siRNA 6	cAAcAuGAuGGcAAGGuAuTsT AuACCUUGCcAUcAUGUUGTsT
siRNA 7	uGGuGuGGAAuAAuGuuAATsT UuAAcAUuAUUCcAcACcATsT
siRNA 8	GGuccuAAAuGGuuGAAGATsT UCUUcAACcAUUuAGGACCTsT
siRNA 9	AuGGcAAGGuAuGAAccAATsT UUGGUUcAuACCUUGCcAUTsT
siRNA 10	cuAAAuGGuuGAAGAAcAcTsT GUGUUCUUcAACcAUUuAGTsT
Control siRNA	cuUaCgCuGaGuAcUuCgATCGAAGTATsT UCgAaGuAcUcAgCgUaAgTsT
ASO 1	gsgsgsasusCsCsTsGsGsAsAsAsAsAsAsAsasgsasasu
ASO 2	gsasgsususGsGsAsAsAsGsUsGsAsAscscscsasu
ASO 3	ususgscscsAsTsCsAsTsGsTsTsGsTsascscsusg
ASO 4	cscscscsusCsTsCsAsGsTsGsCsTsGsgsasascsc
ASO 5	csasgscsasTsAsTsCsAsGsCsCsAsAsGscsuscsusg
ASO 6	gsasusasgsGsTsCsAsGsGsGsCsAsGsGsAsususcscsu
ASO 7	ususasgsgsTsGsGsCsAsGsTsTsCsAsgsgsasgsa
ASO 8	gsuscsgsgsTsAsTsCsAsGsTsTsGsCsasgsasgsa
ASO 9	asasgsasasAsAsGsAsTsCsTsTsCsAstsgsgsgsu
ASO 10	gsgststsgsTsTsTsAsCsTsGsAsCsTsTstsgstststsa
ASO 11	csasasgsasTsAsTsCsGsAsTsCsAsGsCsGsususasusa
ASO 12	asasusususGsCsTsGsAsAsGsTsGsTsgsascsgsu
ASO 13	usgsasgsgscsCsCsAsGsTsCsAsGsTsCsCsCsusgscsusasc
ASO 14	usgsususgsusAsCsCsTsGsGsCsCsAsGsTscsasgscsusgsc
Control ASO	tscsgsasasgsTsAsCsTsCsAsGscsgstsasasg

*Note.*Capital letters indicate ribonucleotides, capital italics denote 2’-deoxynucleotides, lowercase letters indicate 2’-O-methylribonucleotides, s stands for thiophosphate groups.

**Transfection of AML12 cells with siRNAs and antisense oligonucleotides**

Small interfering RNAs (siRNA) and antisense oligonucleotides (ASO) (table) were synthesized using the phosphoramidite method and purified by ion exchange chromatography. Oligonucleotide purity was confirmed by LC-MS. AML12 cells (~1 × 10^5^) were transfected with siRNA or ASO at concentrations of 10 and 5 nM, respectively, using the Lipofectamine- RNAiMAX reagent (Invitrogen) according to the manufacturer’s instructions. Small interfering RNA and ASO for luciferase gene were used as a control (table). To obtain the siRNA and ASO mixtures, the components were mixed in an equimolar ratio. Small interfering RNA: No. 1: 1+2+3+6; No. 2: 1+3+2; No. 3: 1+3+6; No. 4: 1+3+6; No. 5: 2+3+6. ASO: No. 1: 3+4+7+8+14; No. 2: 3+7+14; No. 3: 3+4+14; No. 4: 3+14. Total RNA was isolated 24 h after transfection using the Trizol reagent (Invitrogen, USA) according to the manufacturer’s protocol. Knockdown efficiency was analyzed by RT-qPCR.

**Western blot**

The AML12 cells (~1×10^6^) were lysed in RIPA buffer (Thermo Scientific, USA) containing 1 mM DTT, 0.05% Triton X-100, 0.2 mM phenylmethylsulfonyl fluoride, protease and phosphatases inhibitors. The concentration of proteins in lysates was determined by spectrophotometry using the Bradford reagent (Thermo Scientific, USA). For further analysis, a lysate containing 40 μg of the protein, preliminarily denatured at 95°C for 5 min, was used. The proteins were separated in 10% PAGE in Tris-glycine buffer (pH 8.3), and then they were transferred to a PVDF membrane (Millipore) at a voltage of 70 V for 1 h. Next, the membrane was incubated with a 5% BSA solution for 1 h, and then with a solution of specific antibodies. The antibodies against Foxa2 (ab108422 Abcam, USA) and actin (ab179467 Abcam, USA) proteins, horseradish peroxidase conjugates with antibodies against rabbit and mouse immunoglobulins (ab6721 and ab6728 Abcam, USA) were used. Secondary antibodies were visualized on the membranes by chemiluminescence using a Pierce ECL kit (Thermo Scientific, USA) according to the manufacturer’s protocol.

**Statistical analysis of the experimental data**

All the diagrams are based on at least three independent experiments. Statistical data processing was performed using the GraphPad Prism software (version 6.0) with a two-sample t-test, as well as a two-way ANOVA analysis of variance or repeated-measures ANOVA and Sidak t-test. The data were considered statistically significant at P < 0.05.

**Prediction of LL35 lncRNA secondary structure**

To propose secondary structure of the LL35 lncRNAs, the ViennaRNA software package (http://rna.tbi.univie.ac.at/) was used. It predicts secondary RNA structures with minimal free energy taking into account the probability of base pair formation for RNA.

## RESULTS

**Determination of the LL35 lncRNA expression level in mouse organs and AML12 liver cells**

**Fig. 1:**
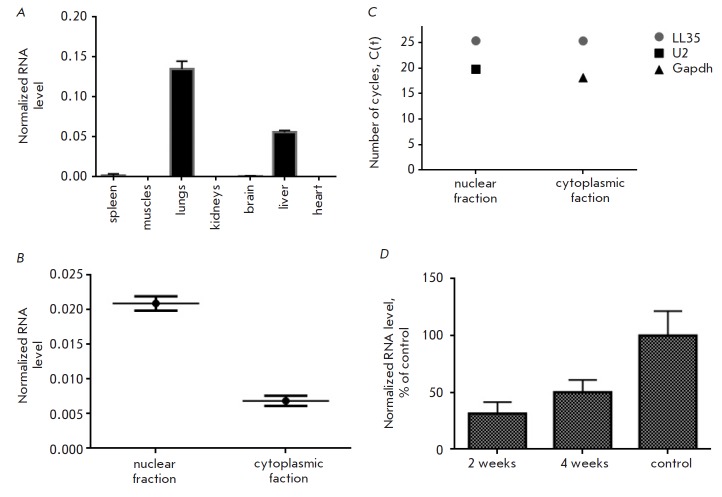
A) Quantification of LL35 lncRNA in mouse organs (control – Gapdh mRNA); B) Determination of the cellular localization of LL35 in AML12 hepatocytes (controls – U2 RNA for the nuclear fraction, Gapdh mRNA for the cytoplasmic fraction); C) Comparison of LL35 RNA distribution between the nuclear and cytoplasmic fractions of AML12 cells using U2 and Gapdh RNAs as a reference; D) Quantification of LL35 lncRNA in the samples of mouse liver with CCl4-induced fibrosis at weeks 2 and 4 (control – Gapdh mRNA)

According to NCBI database, the LL35 lncRNA gene (9020622O_2_2Rik) is located 2,500 nucleotides downstream of the Foxa2 transcription factor gene and encodes 38 annotated transcripts, the majority of which share the same exons (the first and the last two ones) (https://www.ncbi.nlm.nih.gov/gene/?term=9030622O22Rik). Analysis of the expression level of exons common to all transcripts was used to determine the level of LL35 lncRNA in the mouse organs. Tissue- specific expression and the highest abundance of LL35 lncRNA were shown for lungs and the liver (Fig. 1A). To determine the localization of LL35 lncRNA in normal liver AML12 cells, nuclear and cytoplasmic cell fractions were isolated for further analysis by RT-qPCR. LL35 lncRNA is predominantly located in the cell nucleus: only ~20% of the total RNA is located in the cytoplasm (Fig. 1B). Moreover, the level of LL35 RNA in the nucleus is only 1.3 times lower than that of U2 snRNA, which is an indication of a high transcription level for lncRNA (Fig. 1C).

It has been previously shown that the level of the transcription factor Foxa2 mRNA decreases in liver fibrosis, while a deletion of this factor predisposes one to the development of fibrosis [19]. We noted a up to 70% decrease in LL35 lncRNA in mouse liver two weeks after fibrosis induction with carbon tetrachloride, followed by partial restoration of lncRNA levels up to 60% of their base level four weeks after induction (Fig. 1D).

**Selection of the conditions for LL35 lncRNA knockdown in AML12 cells**

**Fig. 2:**
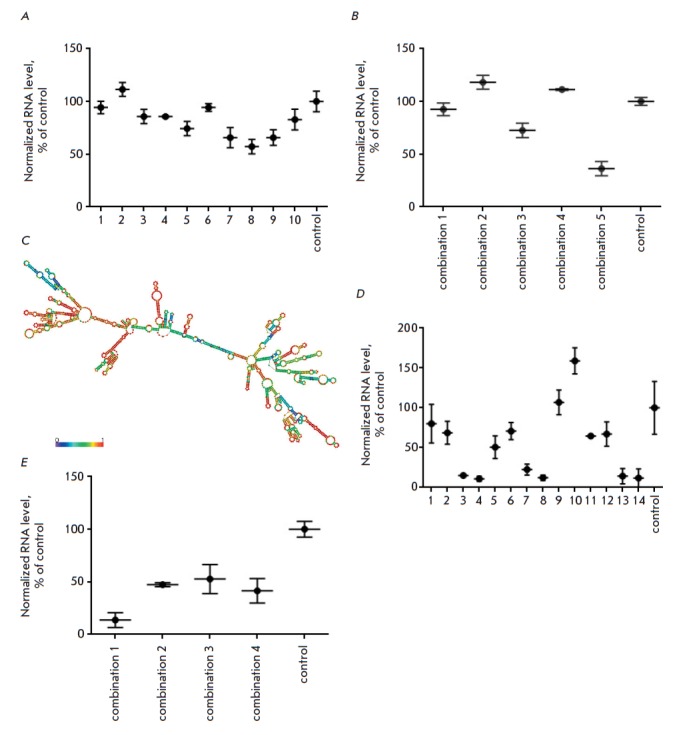
Knockdown of LL35 lncRNA in AML12 mouse hepatocytes by: A) individual siRNAs (No. 1–10; control – siRNA for firefly luciferase mRNA); B) combinations of siRNAs (No. 1–5; control — siRNA for the firefly luciferase gene); C) A model of LL35 lncRNA; color scale indicates the probability of formation/stability of the secondary structure elements; D) Individual antisense oligonucleotides (No. 1–14; control — ASO for the firefly luciferase gene); E) combinations of ASO (No. 1–4; control — ASO for the firefly luciferase gene)

At the first stage of the study, we used RNA interference to knockdown LL35 RNA in vitro. The sequence of LL35 RNA was analyzed, and possible siRNA binding sites, as well as sequences found in other RNAs of the mouse transcriptome, were excluded. Afterwards, 10 siRNAs specific to LL35 RNA and containing 2’-O-methyl pyrimidine nucleotides and phosphorothioates groups for increasing the stability to intracellular nucleases were designed and synthesized. The efficiency in LL35 knockdown by individual siRNAs did not exceed 40% (Fig. 2A). In order to improve that knockdown efficiency, siRNA combinations (three to four siRNAs in the mixture) were tested. In this case, knockdown achieved approximately 60% of the base LL35 RNA level (Fig. 2B). One of the possible explanations for such a low efficiency of the siRNAs can be the predominant nuclear localization of LL35 lncRNA and the absence of active nucleus ↔ cytoplasm transport.

Taking into account the nuclear localization of LL35 lncRNA, antisense oligonucleotides (ASO) were chosen as an alternative approach to knockdown LL35 lncRNA. A model of the secondary structure of LL35 lncRNA was obtained using the ViennaRNA software for the design of ASO (Fig. 2C) [20]. Fourteen antisense oligonucleotides complementary to the helixes in the predicted RNA structure were selected. ASO 3, 4, 7, 8, 13, and 14 showed a higher efficiency for LL35 lncRNA knockdown (Fig. 2D) than individual siRNAs and their combinations. The use of ASO combination No. 1 allowed us to reduce the level of LL35 lncRNA expression to 90% of the basal level. Therefore, this combination was selected for further studies (Fig. 2E).

**LL35 lncRNA is involved in the regulation of the Foxa2 transcription factor in AML12 mouse hepatocytes**

**Fig. 3:**
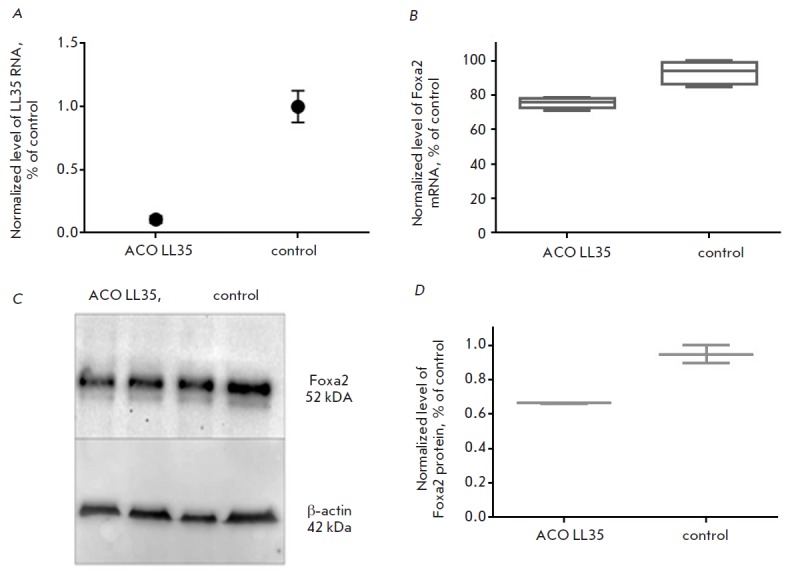
Efficacy of LL35 lncRNA knockdown in AML12 mouse hepatocytes with ASO mix No. 1; B) knockdown of LL35 RNA results in downregulation of the transcription factor Foxa2 mRNA; C), D) Analysis of the Foxa2 protein level under conditions of LL35 RNA knockdown (control — β-actin)

Previously, analysis of the transcriptome of human embryonic stem cells at the differentiation stage revealed a correlation between changes in the expression of both DEANR1 lncRNA and Foxa2 mRNA. A possible mechanism for the activation of the transcription of Foxa2 through Smad2/3 transcription modulators that directly interact with DEANR1 has been proposed [14]. The level of Foxa2 mRNA decreases by 20% of the basal mRNA level (Fig. 3B) upon knockdown of the functional analogue of DEANR1, LL35 lncRNA, in mouse hepatocytes (Fig. 3A). The level of the Foxa2 protein is reduced by 30% (Fig. 3C).

## DISCUSSION

The Foxa2 transcription factor is an important regulator of endoderm differentiation into various types of tissues. In an adult liver, Foxa2 is required for normal functioning of the organ and acts as one of the main regulators of the transcription of the liver-specific genes encoding key participants in the lipid metabolism and ketogenesis [16]. The important role of Foxa2 in regulating liver organogenesis should be also noted [21]. In genome, human lncRNA DEANR1 and mouse lncRNA LL35 are located in close proximity to the Foxa2 gene. A mechanism has been proposed for the regulation of the Foxa2 by human lncRNA DEANR1 in embryonic stem cells [14]. The genomic localization of the LL35 lncRNA gene suggests that LL35 lncRNA, which can be involved in the regulation of the transcription factor Foxa2, performs similar functions.

According to the data in the NCBI database, the hierarchy of abundance of human DEANR1 lncRNA is as follows: in the liver, then the stomach, then in the lungs, pancreas, and intestines (https://www.ncbi.nlm.nih.gov/gene/140828). Analysis of the level of a potential functional analogue of DEANR–LL35 RNA–in mouse organs revealed its tissue-specific expression in the lungs and liver, which is slightly different from the expression of human DEANR1 RNA. It is possible that there are differences in the function of DEANR1 and LL35 in some organs. Therefore, we selected the liver as our object of further studies, since both lncRNAs are highly abundant in the liver.

We have shown that, in mouse hepatocytes, LL35 lncRNA, as well as human DEANR1 lncRNA, is located mainly in the nucleus. The use of antisense oligonucleotides is considered to be the optimal approach to knockdown and study of the function of lncRNAs that have targets localized in the nucleus. However, siRNA can be also used in case of active nucleus ↔ cytoplasm transport. Small interfering RNAs are advantageous for in vivo studies, because they are effective at lower doses and provide a longer suppression of the target expression while minimizing hepatotoxicity. Having compared the two approaches, we have chosen ASOs, which knockdown LL35 lncRNA with higher efficiency. However, the use of individual ASO to suppress LL35 lncRNA can result in the preservation of the functionally active part of lncRNA. In order to increase the probability of inactivation of the functional center of LL35 lncRNA, the efficiency of LL35 knockdown by various ASO combinations was tested. Using this approach, we were able to achieve a 90% knockdown of LL35 lncRNA in mouse hepatocytes. Knockdown of the lncRNA in AML12 mouse hepatocytes resulted in a 20% decrease of Foxa2 mRNA and 30% decrease of the Foxa2 protein. The obtained data are in good agreement with the published ones on the changes in the Foxa2 level in mouse lung cells in embryonic knockout of the LL35 lncRNA gene. We suggest a similar mechanism of regulation for the transcriptionfactor Foxa2 through recruitment of Smad2/3 proteins to the Foxa2 promoter region resulting in transcription activation [14]. One can also assume that, in an adult healthy liver, LL35 lncRNA is involved in maintenance of normal liver function by regulating the activation of Foxa2 and its targets, depending on external signals.

LncRNA functions and the molecular mechanisms they are involved in are studied mainly in vitro, which complicates determination of their role in the development of various diseases. The optimal approach to solving this problem is studying lncRNA functions in vivo. It has been previously shown that, in liver fibrosis, the transcription factor Foxa2 is suppressed, which results in stress to the endoplasmic reticulum and leads to the death of hepatocytes [19, 22]. The level of Foxa2 also decreases in hepatic injuries of various etiologies [22]. The decrease in the LL35 lncRNA level detected in liver samples with induced fibrosis is consistent with previously published data on a decrease in the level of Foxa2 mRNA, which also confirms the existence of the regulation. Moreover, the expression level of LL35 lncRNA on the second and fourth week after fibrosis induction is consistent with the proliferative activity of hepatocytes during liver regeneration in fibrotic damage [23].

## CONCLUSIONS

Based on the data obtained, as well as previously published data, we can conclude that mouse LL35 lncRNA is a functional analogue of human lncRNA DEANR1. In the present work, tissue-specific expression of LL35 lncRNA has been demonstrated in the liver and lungs. Nuclear localization was established for liver cells, and efficient knockdown of LL35 lncRNA expression by ASO was demonstrated for the first time. A decrease in the level of LL35 RNA results in a decrease in the level of Foxa2 mRNA and protein in liver cells. For the first time, a decrease in the level of LL35 lncRNAs in liver fibrosis was determined, which indicates the potential of further studies of lncRNA in vivo. Based on the obtained results, one can assume that LL35 lncRNA is involved in the molecular mechanisms of endoplasmic reticulum stress in hepatocytes, which occurs in liver fibrosis. On the other hand, LL35 lncRNA can also contribute to fibrosis via interaction with Smad2/3 transcription modulators in stellate cells [14]. For example, LFAR1 lncRNA regulates the level and degree of phosphorylation of Smad2/3 proteins, which, in turn, causes their translocation to the nucleus and activates the expression of a number of genes, including those involved in the synthesis of type I collagen [24]. All these hypotheses require further confirmation, while the possibility of targeted in vivo delivery of the proposed antisense oligonucleotides to liver cells allows one to study LL35 lncRNA in various mouse models of liver diseases [25].

## Abbreviations

lncRNA: long non-coding RNA
ASO: antisense oligonucleotides

## References

[1] Al-Tobasei R., Paneru B., Salem M. (2016). PLoS One..

[2] Long Y., Wang X., Youmans D.T., Cech T.R. (2017). Sci. Adv. 2017. V. 3. № 9. P. eaao2110..

[3] Dykes I.M., Emanueli C. (2017). Genom. Proteom. Bioinf..

[4] Tripathi V., Ellis J.D., Shen Z., Song D.Y., Pan Q., Watt A.T., Freier S.M., Bennett C.F., Sharma A., Bubulya P.A. (2010). Molecular Cell.

[5] Militello G., Weirick T., John D., Döring C., Dimmeler S., Uchida S. (2017). Brief. Bioinform..

[6] Smekalova E.M., Kotelevtsev Y.V., Leboeuf D., Shcherbinina E.Y., Fefilova A.S., Zatsepin T.S., Koteliansky V. (2016). Biochimie..

[7] Wang Y., Xue K., Guan Y., Jin Y., Liu S., Wang Y., Liu S., Wang L., Han L. (2017). Oncol. Res..

[8] Zhang H.F., Li W., Han Y.D. (2018). Cancer Biomark. Sect. Dis. Markers..

[9] Wang Y., Xue K., Guan Y., Jin Y., Liu S., Wang Y., Liu S., Wang L., Han L. (2017). Oncol. Res..

[10] Fan Y., Wang Y.F., Su H.-F., Fang N., Zou C., Li W.-F., Fei Z.H. (2016). J. Hematol. Oncol..

[11] Müller S., Raulefs S., Bruns P., Afonso-Grunz F., Plötner A., Thermann R., Jäger C., Schlitter A.M., Kong B., Regel I. (2015). Mol. Cancer..

[12] Wang Z.K., Yang L., Wu L.L., Mao H., Zhou Y.H., Zhang P.F., Dai G.H. (2017). Braz. J. Med. Biol. Res..

[13] Liu Y., Xiao N., Xu S.F. (2017). Eur. Rev. Med. Pharmacol. Sci..

[14] Jiang W., Liu Y., Liu R., Zhang K., Zhang Y. (2015). Cell Rep..

[15] Lee C.S., Friedman J.R., Fulmer J.T., Kaestner K.H. (2005). Nature.

[16] Wolfrum C., Asilmaz E., Luca E., Friedman J.M., Stoffel M. (2004). Nature.

[17] Herriges M.J., Swarr D.T., Morley M.P., Rathi K.S., Peng T., Stewart K.M., Morrisey E.E. (2014). Genes Dev..

[18] Swarr D.T., Herriges M., Li S., Morley M., Fernandes S., Sridharan A., Zhou S., Garcia B.A., Stewart K., Morrisey E.E. (2019). Genes. Dev..

[19] Wang W., Yao L.J., Shen W., Ding K., Shi P.M., Chen F., He J., Ding J., Zhang X., Xie W.F. (2017). Sci. Rep..

[20] Lorenz R., Bernhart S.H., Höner zu Siederdissen C., Tafer H., Flamm C., Stadler P.F., Hofacker I.L. (2011). Algorithms Mol. Biol..

[21] Lee C.S., Friedman J.R., Fulmer J.T., Kaestner K.H. (2005). Nature.

[22] Wang K. (2015). Cell. Signal..

[23] Koyama Y., Brenner D.A. (2017). J. Clin. Invest..

[24] Peng H., Wan L.Y., Liang J., Zhang Y.Q., Ai W.B., Wu J.-F. (2018). Cell Biosci..

[25] Crooke S.T., Witztum J.L., Bennett C.F., Baker B.F. (2018). Cell Metab..

